# Decoding Cancer Risk: Understanding Gene-Environment Interactions in Cancer Development

**DOI:** 10.7759/cureus.64936

**Published:** 2024-07-19

**Authors:** Ajay Pal Singh Sandhu, Tanvir, Kanwarmandeep Singh, Sumerjit Singh, Harman Antaal, Shivansh Luthra, Abhinandan Singla, Gurkamal Singh Nijjar, Smriti K Aulakh, Yasmeen Kaur

**Affiliations:** 1 Internal Medicine, Sri Guru Ram Das University of Health Sciences and Research, Amritsar, IND; 2 Medicine, Government Medical College Amritsar, Amritsar, IND; 3 Internal Medicine, Government Medical College Amritsar, Amritsar, IND; 4 Internal Medicine, Government Medical College Patiala, Patiala, IND

**Keywords:** environmental carcinogenesis, sleep patterns and cancer risk, diet and physical activity in cancer prevention, infectious agents in carcinogenesis, radiation-induced cancers, lifestyle and oncology, pollution and cancer, gene-environment interactions, cancer risk factors, immune system abnormalities

## Abstract

While lifestyle choices or behavioral patterns remain the most significant factors influencing cancer risk, environmental exposure to certain chemicals, both manufactured and natural, may also contribute to an individual's likelihood of developing cancer. This interplay of factors, coupled with an aging demographic and shifting lifestyle patterns, has led to an increasing prevalence of cancer in recent years. This study examines the environmental and behavioral factors that contribute to anomalies in the immune system and increase the risk of developing cancer. Significant environmental and occupational factors include the contamination of air and water, exposure to radiation, contact with harmful microorganisms and pathogens, and workplace exposure to carcinogens such as asbestos, certain chemicals, and industrial pollutants. Behavioral factors, such as food, physical activity, stress, substance misuse, and sleep patterns, have a substantial impact on immunological function and the likelihood of developing cancer. For example, pollutants like benzene and arsenic can disrupt immune function and raise the risk of developing cancer. Similarly, lifestyle variables such as inactivity and poor nutrition have been linked to an increased risk of cancer. Long-term stress and substance abuse can also decrease immunological responses, increasing the risk of developing cancer. The review underlines the complexities of examining gene-environment interactions, as well as the importance of using several perspectives to fully comprehend these pathways. Future investigations should emphasize improved methodology and larger sample sizes. Public health campaigns should aim to reduce human exposure to cancer-causing compounds known as carcinogens while also encouraging the adoption of healthy behaviors and habits. Tailored preventive approaches that account for individual genetic vulnerabilities have the potential to improve cancer prevention and treatment.

## Introduction and background

In 2022, 20 million new cancer cases were reported, with 9.7 million cancer-related deaths. Within five years of receiving a cancer diagnosis, approximately 53.5 million people survived. Approximately one in every five people will develop cancer in their lifetime, with one in every nine men and one in every 12 women dying from the disease [1]. In 2023, the United States reported 1,958,310 new cancer cases and 609,820 cancer deaths [2]. Cancer rates are increasing due to a multitude of factors, including aging populations, lifestyle and behavioral changes, and environmental exposures [3-5]. Cancer has far-reaching consequences, affecting not just the people who have been diagnosed but also their families, healthcare institutions, society as a whole, and the broader economy through healthcare costs and low productivity. Cancer burden reduction requires effective prevention, early detection, and treatment options.

Cancer is defined by unregulated cell division and replication as a result of regulatory pathway breakdown. This disturbance can be caused by environmental, behavioral, or genetic variables, which activate oncogenes and deactivate tumor suppressor genes, resulting in various cancer types [5] (Table 1). Environmental variables account for more than half of cancer cases, emphasizing the relevance of modifiable exposures.

**Table 1:** Risk factors associated with different types of cancers

Cancer type	Risk factor
Melanoma	Weakened immune system
	Dysplastic nevi
	Fair skin
	Sunburn/severe blistering
	UV irradiation
Uterine cancer	Endometrial hyperplasia
	Hormonal replacement therapy
	Obesity
	Race: African Americans
Bladder cancer	Tobacco smoking
	Certain infection
	Occupation
	Race: Twice as often as African Americans
	Treatment with cyclophosphamide or arsenic
Liver cancer	Hepatitis viruses (HCV, HBV)
Kidney cancer	Tobacco smoking
	High blood pressure
	von Hippel-Lindau (VHL) syndrome
Pancreatic cancer	Diabetes
	Smoking
	Male sex
	Chronic pancreatitis
Leukemia	Radiation exposure
	Air pollution (benzene)
	Chemotherapy
	Certain diseases (Down syndrome)
	Human T-cell leukemia virus
	Myelodysplastic syndrome
Lung cancer	Tobacco smoking
	Radon exposure
	Asbestos and other substances
	Air pollution
Colorectal cancer	Cancer polyp
	Genetic alteration
	Diet
	Cigarette smoking
	Ulcerative colitis/Crohn's disease
Breast cancer	Radiation exposure
	Inherited mutation
	Dietary cadmium
Prostate cancer	Diet
	Certain prostate changes
	Race: African Americans

**Figure 1:**
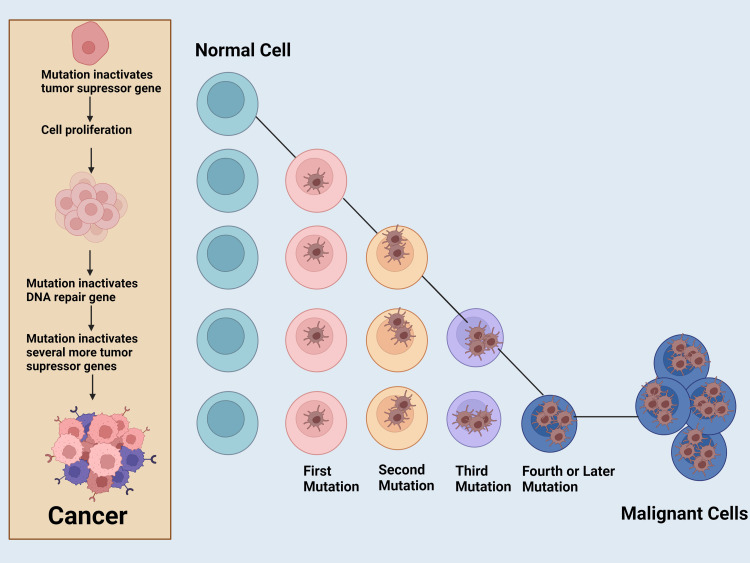
Cancer mutagenesis The initial mutation in a healthy cell inactivates a negative cycle regulator, the next mutation overactivates a positive cell cycle regulator, the third mutation inactivates a genome stability factor, and additional mutations accumulate rapidly resulting in cancer cells. The figure has been created in BioRender.com

There are three stages of carcinogenesis: initiation, promotion, and progression. It includes exposure to carcinogens that lead to genomic instability and potentially irreversible mutations [6] (Figure 1). When the promotion phase alters the surrounding cell population to support neoplastic proliferation, progression leads to a more aggressive/malignant function. With an increase in the early detection of cancer and medical advances that have led to decreased mortality from some cancers, more studies are required to assess bidirectional relationships among multiple risk factors present during cancer development.

This review examines the environmental and behavioral factors that contribute to immune system abnormalities, as well as the consequences for the risk and prevention of oncopathologies. We investigated how pollution, radiation exposure, and climate change affect immune function. Behavioral issues such as food, physical exercise, stress, substance abuse, and sleep habits, as well as their impact on immunological health and cancer risk, were also discussed. By integrating current information and highlighting knowledge gaps, this review seeks to provide a thorough understanding of how these factors lead to immune system aberrations and oncopathologies. Finally, we want to draw attention to potential preventative and intervention techniques that may reduce the risk of acquiring cancer.

## Review

Environmental contributors to immune system abnormalities and oncopathologies

The relationship between environmental factors and immune system abnormalities is a critical area of research, particularly concerning the risk of malignancies. Various environmental contributors, such as pollution, radiation exposure, climate change, microbial and pathogen exposure, and occupational exposure to carcinogens and hazardous substances, play a significant role in modulating the immune system and potentially increasing the risk of carcinomas [7].

Pollution and Toxins

Air pollution, particularly exposure to particulate matter (PM), benzene, and other volatile organic compounds, has been linked to immune system dysregulation. Benzene is a well-known carcinogen in humans. The International Agency for Research on Cancer (IARC) has categorized benzene as a group 1 carcinogen, signifying a well-established relation to human cancer [7]. There is enough evidence to suggest that benzene exposure causes acute nonlymphocytic leukemia, specifically acute myeloid leukemia [8]. However, the evidence linking benzene to non-Hodgkin's lymphoma (NHL), chronic lymphoid leukemia, multiple myeloma, chronic myeloid leukemia, acute myeloid leukemia in children, and lung cancer is limited [9]. Furthermore, benzene has been found to cause a variety of malignancies in laboratory animals, including offspring of exposed females. Benzene-induced hematotoxicity is linked to an increased chance of developing hematological cancer or similar illnesses [10].

Water contamination is induced by a variety of mechanisms, including microorganism infection (bacteria, viruses, protozoa, and parasitic worms). Wastes degraded by oxygen-requiring bacteria result in fish death due to oxygen decrease. Acids, salts, and poisonous metals kill aquatic life. Nutrients such as water-soluble nitrates and phosphates induce algae to develop excessively by consuming oxygen from the water, resulting in fish death [11]. Water, polluted by different organic substances such as oil, plastics, detergents, chloroform, petroleum, polychlorinated biphenyl (PCB), fertilizer, sulfur oxide, pesticides, and trichloroethylene and arsenic, poses significant health risks. Industrial chemicals like PCBs and per- and polyfluoroalkyl substances (PFAS) have been implicated in immune dysfunction and increased lymphoma risk [12]. Arsenic, found in contaminated drinking water, can cause chronic immune suppression and has been linked to various cancers including skin cancer [13].

Iwasaki et al. examined epidemiological data on cancer risk and exposure to dichlorodiphenyltrichloroethane (DDT), hexachlorocyclohexane (HCH), PCBs, PFASs, cadmium, arsenic, and acrylamide in the Japanese population [12]. Japanese people are heavily exposed to these substances, primarily through their cuisine, and a link with an increased cancer risk is believed. Epidemiological evidence from Japanese research to date does not show a link between blood concentrations of DDT, HCH, PCBs, and PFASs and the risk of breast or prostate cancer. Using a food frequency questionnaire, we developed methods for estimating cadmium, arsenic, and acrylamide dietary intake. Overall, the Japan Public Health Center-based Prospective Study found no significant link between cadmium, arsenic, and acrylamide food intake and an elevated risk of overall cancer or major cancer sites. However, there were statistically significant positive relationships between dietary cadmium intake and the risk of estrogen receptor-positive breast cancer in postmenopausal women, as well as dietary arsenic intake and the risk of lung cancer in male smokers. Furthermore, research using biomarkers to evaluate exposure indicated statistically significant positive relationships between urine cadmium content and risk of breast cancer, as well as between acrylamide-glycidamide hemoglobin adduct ratio and risk of breast cancer [13].

In terms of cancer, several authors point out that farm workers are less likely to develop cancer than the general population, most likely due to a lower intake of alcohol and drugs and a higher level of physical activity (PA) in their professional practices [14]. However, some types of cancer are more common in this population, either as a result of ultraviolet ray exposure, as in the case of lip cancer and myeloma, or pesticide exposure, which is linked to an increased risk of prostate and hematological cancer, as well as NHL and even brain tumors [15-17]. A research on pesticide exposure and bladder cancer found no definitive link between these variables, but it did show a significantly greater prevalence of cancer in vegetable producers, women, and nonsmokers [17].

Koutros et al. identified occupational pesticide exposure as the primary factor contributing to the risk of bladder cancer [18]. Lesseur et al. [19] performed a study in New Hampshire to examine the interactions between genes and the environment. Specifically, they investigated the effects of genetic variations and low levels of arsenic exposure on a community. The researchers highlighted that the likelihood of developing bladder cancer may be linked to the genetic variation in a gene responsible for arsenic metabolism and oxidative stress [19]. Additionally, the study discovered that persons with the AQP3 genotype had a higher likelihood of developing bladder cancer in the high arsenic exposure group, compared to those in the low arsenic exposure group who had the same genotype. Various carcinogens in the workplace have been listed in Table 2.

**Table 2:** Various carcinogens at the workplace and associated risk of cancer type

Carcinogen	Occupation	Type of cancer
Arsenic	Mining, pesticide workers	Lung, skin, liver
Asbestos	Construction workers	Lung, mesothelioma
Benzene	Petroleum, rubber, and chemical workers	Leukemia
Chromium	Metal workers, electroplaters	Lung
Leather dust	Shoe manufacturing	Nasal, bladder
Radon	Underground mining	Lung
Soots, tars, oils	Coal, gas, and petroleum workers	Lung, skin, liver
Vinyl chloride	Rubber workers, polyvinyl chloride manufacturing	Liver
Wood dust	Furniture manufacturing	Nasal

Radiation Exposure

Over the last five decades, epidemiology has greatly improved our understanding of the cancer risks linked with radiation exposure. Epidemiological research has supplied crucial data for assessing the dangers associated with medical, occupational, and environmental exposures, as well as for developing radiation protective regulations [20]. The data from Japanese A-bomb survivors are the principal source for evaluating carcinogenic hazards from low linear energy transfer (LET) external exposure [21]. Furthermore, studies involving persons exposed for medical reasons provide useful additional data, particularly for fractionated high-dose exposure [22]. While radiation exposure has been related to the majority of solid tumors, the most credible risk estimates are for leukemia, all combined solid malignancies, and breast and thyroid cancers [20-21]. Furthermore, multiple cohorts of underground miners and case-control studies of those exposed to radon in their homes have allowed for reasonable estimates of the risks of lung cancer from radon progeny exposure [23-24].

Microbial and Pathogen Exposure

**Figure 2:**
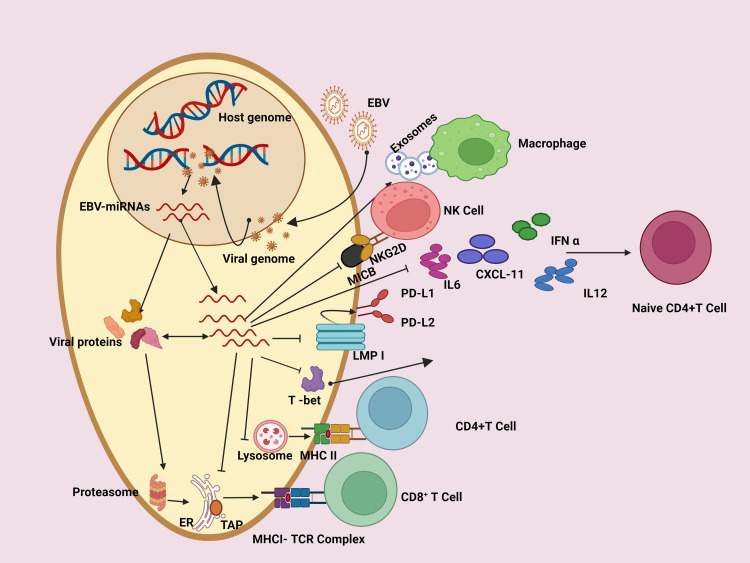
Role of EBV miRNA in regulating the host immune response The biogenesis of EBV-encoded miRNA is dependent on host processes and regulates the antiviral adaptive immune response of infected B cells. Immediately following infection, the viral DNA genome is circularized, and virally encoded and non-coded RNA is generated. EBV miRNA promotes immune evasion on numerous mechanisms. EBV: Epstein-Barr virus; CXCL-11: C-X-C motif chemokine ligand 11; ER: endoplasmic reticulum; TCR: T-cell receptor; MHC: major histocompatibility complex; NKG2D: natural killer group 2D; MICB: MHC class I chain-related molecule B; IL-6R: interleukin-6 receptor; NK cell: natural killer cell; PD-L1: programmed cell death ligand 1; PD-L2: programmed cell death ligand 2; LMP: low malignant potential; IL12: interleukin 12; T-bet: T-box transcription factor 21; TAP: transporter associated with antigen processing

Certain viruses and bacteria can affect the immune system (Figure 2). Infectious microorganisms are believed to be responsible for 18% of all cancer cases worldwide, with poor countries bearing a substantially larger burden (26%) than developed ones (8%) [25]. Human papillomaviruses (HPVs) are the leading cause of cervical cancer, and infection with an oncogenic strain of HPV is required for its development, with vaccination considerably lowering precancerous lesions [26]. Both the hepatitis B and C viruses are known to cause liver cancer [27]. Human T-cell leukemia/lymphoma virus-1 (HTLV-1) increases the risk of lymphoma and leukemia, whereas human immunodeficiency virus (HIV), which causes acquired immunodeficiency syndrome (AIDS), dramatically increases the risk of lymphoma and Kaposi's sarcoma [28]. Epstein-Barr virus (EBV) is associated with Burkitt's lymphoma, and human herpesvirus-8 (HHV8) is a key risk factor for Kaposi's sarcoma [28]. *Helicobacter pylori*, which causes stomach ulcers, is linked to MALT lymphoma and esophageal cancer [29]. Furthermore, *Salmonella typhi* and *Streptococcus bovis* are linked to gallbladder and colon cancer, respectively [30].

According to the hygiene hypothesis, a lack of exposure to infectious organisms during early life can cause immunological dysregulation and an increased risk of autoimmune and allergy illnesses [31]. This lack of immune system "training" may potentially predispose people to cancer later in life as a result of poor immunological responses to infections and other stressors [13].

Climate Change

Climate change can have an impact on cancer development and treatment, both directly and indirectly. Climate change-induced disasters may expose people to carcinogenic substances in the environment, such as formaldehyde and benzene, which are typically found in wildfire smoke [32]. Severe weather might also hamper cancer prevention efforts. Droughts and floods, for example, can reduce food supply and make fresh vegetables more difficult to obtain. Similarly, prolonged heat waves or storms can inhibit outdoor PA.

An eminent study investigating the effects of climate change on cancer focused exclusively on people with lung cancer who were getting radiation therapy [33]. The 2019 study found that those receiving medical treatment in hurricane-affected areas have worse overall survival rates, owing to treatment delays caused by the natural disaster. Furthermore, protracted droughts and heat waves have increased the risk and extent of large-scale wildfires in the Western United States. According to studies, firefighters who are exposed to smoke from these occurrences may have an increased risk of acquiring lung cancer [34]. Wildfire smoke contains fine PM, comparable to the particles found in car exhaust and fossil fuel smoke, which can be harmful to lung and heart health [35].

Behavioral contributors to immune system abnormalities and cancer

Diet and PA

Lifestyle factors such as body weight and PA have an important influence on cancer risk and progression. There is strong evidence that PA lowers and obesity raises the risk and mortality linked with several cancer types [36]. In a variety of preclinical models, energy restriction (ER) in non-obese persons reduces tumor incidence dramatically, and in humans, it lowers body weight and cardiometabolic risk factors. New evidence suggests that the cancer-preventive effects of PA and ER may be mediated by alterations in inflammatory and immunological mediators [37-40]. Both preclinical and clinical investigations show that PA and ER have distinct effects on circulating factors and systemic immune responses. Combining PA and ER can alter the gene expression profile and immune cell infiltrates in tumors, potentially lowering immune suppressive factors. However, further research is needed to properly understand the impact of PA and ER on immunomodulation, particularly in the tumor microenvironment (TME). According to research, women who consume more fried meals tend to have less healthy eating habits, such as higher calorie intake, less PA, and more sedentary behavior. This shows that fried food consumption may be a sign of poor overall eating habits and lifestyle choices. Furthermore, it could interact with genetic predispositions to obesity and higher death rates [41]. Given these findings, women should limit their intake of fried foods, especially given their hereditary vulnerability to obesity, which may lead to cancer development. Interestingly, studies have shown that a high intake of fish and long-chain polyunsaturated fatty acids is associated with genetic changes that may contribute to long-term weight increase in women [42]. In addition to a history of endometriosis, menopausal hormone therapy (MHT), oral contraception, tubal ligation, and breastfeeding habits, exposure to foodborne mutagens is a key risk factor for breast and ovarian cancer development [43].

Breast cancer risk is influenced by gene-environment interactions, such as those involving folate, B vitamins, and polymorphisms in one-carbon metabolism (OCM) genes [44]. Folate in the OCM pathway impacts DNA methylation, synthesis, replication, repair, and gene expression, which all contribute to carcinogenesis. Mutations in OCM genes such as MTHFR 677, MTHFR 1298, and DHFR 19bp impair the folate-mediated pathway, resulting in aberrant DNA methylation and synthesis, which raises the risk of breast cancer [45]. These gene mutations also have an impact on epigenetic changes, inducing hypermethylation of gene loci and the silence of tumor suppressor genes, hence increasing cancer formation [46]. Global hypomethylation, which is associated with aging and genomic instability, also leads to oncogenesis [47].

Stress and Mental Health

The link between stress and cancer is complicated. Chronic stress may predispose people to depression, raising the chance of cancer-related death. A cancer diagnosis, along with the treatment regimens and uncertainties that come with it, is naturally stressful and can have an impact on physical health. An individual's psychological adjustment to cancer may influence long-term outcomes. However, the topic of whether stress contributes to cancer development before diagnosis remains important.

An extensive study has looked into the links between stress-related psychosocial factors and cancer development and outcomes. Chida et al. analyzed 165 research and discovered that stress-related psychosocial factors are associated with a greater risk of cancer in originally healthy populations [4]. Furthermore, an examination of 330 studies found that patients with cancer had lower survival rates when stress-related factors were present, and 53 studies reported a higher cancer mortality rate [4]. Stress-prone personality traits, negative coping mechanisms, and poor emotional responses or quality of life have been linked to increased cancer incidence, lower cancer survival, and higher cancer death. According to specific site analyses, psychological factors are associated with an increased incidence of lung cancer and lower survival rates in patients with breast, lung, head and neck, hepatobiliary, lymphoid, or hematological cancers [48].

Substance Abuse

Lung cancer is the largest cause of cancer-related mortality in the United States [49]. It is seen as a heterogeneous disease, primarily associated with tobacco use. According to research, tobacco smoking accounts for over 60% of the risk of acquiring lung cancer [50]. Cigarettes include around 7000 hazardous chemicals, which are categorized as agents that promote inflammation and cancer in humans [51]. Cigarette smoke exposure is an established risk factor for lung cancer, especially among people living in low-income or underprivileged settings. Furthermore, it has considerable negative economic effects on minority and underrepresented communities.

Furthermore, substance misuse, such as alcohol, tobacco, and recreational drug use, has a major impact on immunological function and raises the risk of lymphoma. Nelson et al. undertook a population-based case-control study to determine if the risk of NHL without HIV infection is connected with prior use of tobacco, alcohol, or recreational substances [52]. The study comprised 378 Los Angeles County residents diagnosed with high- or intermediate-grade NHL, who were compared to individually age-, race-, and gender-matched neighborhood control subjects in terms of tobacco, alcohol, and 10 specific recreational drug usage. Women who had five or more drinks per week had a 50% lower risk of NHL than those who did not drink. Cocaine, amphetamines, Quaaludes, and lysergic acid diethylamide (LSD) have all been related to a significantly higher risk of NHL in men, with increased risk associated with more regular use of these drugs. Confounding variables could not be ruled out in these results. The use of numerous types of medications was also found to be associated with a considerably elevated risk of NHL in men, with the highest risk among those taking five or more types of drugs. According to multivariate analysis, cocaine usage appears to account for the increased risk of NHL among men [52].

Alcohol has been shown by researchers to induce cancer via altering the action of alcohol-metabolizing enzymes such as alcohol dehydrogenase (ADH), acetaldehyde dehydrogenase (ALDH), and cytochrome P450 2E1. It also has an effect on the activity of the methylenetetrahydrofolate reductase (MTHFR) enzyme, which is required for folate metabolism [53]. Another study undertaken by the Breast Cancer Association Consortium identified substantial gene-environment interactions between CFLAR-rs7558475 and cigarette smoking, as well as 5q14-rs7707921 and alcohol intake, in relation to the risk of estrogen receptor-negative breast cancer [54].

Sleep Patterns

Poor sleep has become a major public health issue. Poor sleep has been linked to an increase in sickness risk as sleep duration has reduced. Sleep is essential for physical and mental health [55]. A recent study has looked into the effect of sleep duration on a number of health outcomes, including cancer [56-58]. Epidemiological studies provide valuable information about sleep-health interactions at the community level [59-60]. However, the evidence linking sleep duration to cancer risk is varied, with studies reporting negative [61-62], positive [63-65], and null [66-68] effects. The dose-response relationship across various sleep duration categories remains unclear [68]. These contradictory findings highlight the need for more research to better understand the complex relationship between sleep duration and cancer risk.

Liu et al. employed mass cytometry and single-cell RNA sequencing to map the human immune cell landscape during inadequate sleep [69]. The landscape was then examined in terms of subset composition, gene signatures, enriched pathways, transcriptional regulatory networks, and intercellular connections. Being awake increased the number of T and plasma cells, as well as the expression of autoimmune markers and pathways in CD4+ T and B cells. Furthermore, staying awake reduced the differentiation and immunological activity of cytotoxic cells, indicating an increased risk of infection and tumor growth. Finally, being awake altered the distribution of myeloid subsets, causing inflammation and cellular senescence. These findings may provide high-dimensional and sophisticated insights into the cellular and molecular processes underlying pathologic illnesses associated with insufficient sleep.

Challenges and future directions

Environmental and behavioral aspects are complex, making investigating gene-environment interactions challenging. Because of the variety of environmental components, scientific investigations have been called into question. Furthermore, assessment methodologies can change greatly across different sites, such as homes, communities, and workplaces, resulting in inaccurate estimates and difficulty recognizing consequences. Human exposures and biological consequences also change considerably from conception to adulthood. Environmental and behavioral factors can have overlapping impacts on malignant development, leading to non-linear correlations in gene-environment studies [43,70]. Researchers concur that exposure effects and illness outcomes differ between genotypes and geographic contexts due to geographical, chronological, and social factors [71]. Small sample numbers, ambiguity in quantifying environmental exposures, problems incorporating the quantity of exposure, and variability in gene and environmental factors can all contribute to reported variances. As a result, many environmental exposures are highly associated, making it difficult to isolate the impact of a single exposure [72].

Research methodologies and statistical analysis in genetics and environmental interplay have experienced challenges. Small-scale genetic and environmental analyses can be performed using statistical tools such as SAS, STATA, and SPSS. However, these methods fail to handle big sample sizes and massive amounts of data from genome-wide association studies (GWAS) or more complicated models [73]. Large-scale investigations on genetics and environmental interactions necessitate more sophisticated programming frameworks. As a result, novel programming strategies capable of dealing with large amounts of data must be devised.

## Conclusions

This review highlights the complex interactions between environmental and behavioral factors, immune system abnormalities, and cancer risk. Evidence shows that pollutants, radiation, microbial agents, and lifestyle choices significantly impact immune function and carcinogenesis. Research challenges include exposure complexity and measurement inconsistencies. Future studies require improved methodologies and multidisciplinary approaches to understand these mechanisms. Furthermore, public health initiatives should focus on reducing carcinogen exposure, promoting healthy lifestyles, and improving early detection. Nonetheless, personalized approaches considering genetic susceptibilities and environmental exposures offer promising avenues for cancer prevention and treatment, such as tailored screening protocols based on individual genetic risk factors and targeted therapies designed to address specific molecular alterations in tumors.
